# Risk of data leakage in estimating the diagnostic performance of a deep-learning-based computer-aided system for psychiatric disorders

**DOI:** 10.1038/s41598-023-43542-8

**Published:** 2023-10-03

**Authors:** Hyung-Tak Lee, Hye-Ran Cheon, Seung-Hwan Lee, Miseon Shim, Han-Jeong Hwang

**Affiliations:** 1 Department of Electronics and Information Engineering, Korea University, Sejong, Republic of Korea; 2 Interdisciplinary Graduate Program for Artificial Intelligence Smart Convergence Technology, Korea University, Sejong, Republic of Korea; 3 Psychiatry Department, Ilsan Paik Hospital, Inje University, Goyang, Republic of Korea; 4 Clinical Emotion and Cognition Research Laboratory, Goyang, Republic of Korea; 5 Department of Artificial Intelligence, Tech University of Korea, Siheung, Republic of Korea

**Keywords:** Electroencephalography - EEG, Biomedical engineering

## Abstract

Deep-learning approaches with data augmentation have been widely used when developing neuroimaging-based computer-aided diagnosis (CAD) systems. To prevent the inflated diagnostic performance caused by data leakage, a correct cross-validation (CV) method should be employed, but this has been still overlooked in recent deep-learning-based CAD studies. The goal of this study was to investigate the impact of correct and incorrect CV methods on the diagnostic performance of deep-learning-based CAD systems after data augmentation. To this end, resting-state electroencephalogram (EEG) data recorded from post-traumatic stress disorder patients and healthy controls were augmented using a cropping method with different window sizes, respectively. Four different CV approaches were used to estimate the diagnostic performance of the CAD system, i.e., subject-wise CV (sCV), overlapped sCV (oSCV), trial-wise CV (tCV), and overlapped tCV (otCV). Diagnostic performances were evaluated using two deep-learning models based on convolutional neural network. Data augmentation can increase the performance with all CVs, but inflated diagnostic performances were observed when using incorrect CVs (tCV and otCV) due to data leakage. Therefore, the correct CV (sCV and osCV) should be used to develop a deep-learning-based CAD system. We expect that our investigation can provide deep-insight for researchers who plan to develop neuroimaging-based CAD systems for psychiatric disorders using deep-learning algorithms with data augmentation.

## Introduction

Machine-learning approaches have been widely used to develop neurophysiological feature-based computer-aided diagnosis (CAD) systems to assist the accurate diagnosis of psychiatric patients by reducing the potential errors of the traditional diagnosis based on an interview with clinical experts. Among various neuroimaging modalities, electroencephalogram (EEG) has been widely used to develop a CAD system because EEG-based neurophysiological features can well reflect the abnormal functional traits of psychiatric patients, and some studies showed superior diagnostic performances when differentiating psychiatric patients from healthy controls (HCs)^1,2^. To develop a more reliable EEG-based CAD system with a high diagnostic performance for psychiatric patients, recent studies started to introduce state-of-the-art deep-learning algorithms^3,4^.

Although deep-learning algorithms can improve the diagnostic performance of traditional machine-learning-based CAD systems, it is not easy to well train deep-learning models using EEG data due to scarcity of training data recorded from patients with psychiatric disorders, unlike other research fields such as image and language recognition. However, this restriction can be ameliorated by increasing the amount of given data using data augmentation methods^5,6^. Up to now, various data augmentation methods have been proposed, among which a cropping method has been widely used to increase the amount of time-series EEG data via cropping whole-time-series EEG data into many segments based on a specific window length. For example, a 10-s EEG epoch results in a series of five 2-s segments with a window length of 2 s without overlap, and then each segment is used as an independent trial (sample) to train a deep-learning model as well as estimate a diagnostic accuracy^7,8^. Deep-learning-based approaches using the cropping-based data augmentation have shown comparable or improved diagnostic performances compared to those of traditional machine-learning approaches when differentiating psychiatric patients from HCs^9–11^.

Despite the promising results obtained using the cropping-based data augmentation approach in the deep-learning framework, a cropping method should be cautiously applied to the discrimination of psychiatric patients from HCs because it may cause an inflated diagnostic accuracy due to a data leakage problem if training and test data are not correctly and independently separated from all available data^12^. It is well-documented that trials of each training and test data should be completely separated to avoid a data leakage problem, ultimately resulting in overly optimistic performances^12^. However, some of recent EEG-based CAD studies that used cropping methods for data augmentation did not completely separate training and test data after data augmentation, and thereby leading to overly optimistic results. For example, the EEG data of a single patient is augmented into in a series of sub-trials using a cropping method, and some augmented trials are used as training data while the others are used as test data, resulting in a data leakage problem because the augmented trials are simultaneously used for both training and test data. Note that the augmented EEG trials are fundamentally originated from a single patient with homogenous data characteristics. To accurately estimate the diagnostic performance of a CAD system using cropped trials without the data leakage problem, subject-wise cross-validation (sCV) should be performed instead of trial-wise CV (tCV) after data augmentation based on a cropping method, but which has been generally overlooked in CAD studies up to now.

In the present study, we examined the effect of a data leakage problem caused by using a data augmentation method with inappropriate cross-validation on the diagnostic performance of a machine-learning-based CAD system using the clinical EEG data recorded from 77 post-traumatic stress disorder (PTSD) patients and 58 HCs. The objective of this study was to present the issue of inflated diagnostic performance caused by data leakage in psychiatric disorders and then to provide its solution based on an appropriate CV method. To this end, we computed diagnostic performances using four different types of CV strategies (sCV, overlapped sCV (osCV), tCV, and overlapped tCV (otCV)) where we employed two convolutional neural network (CNN)-based deep-learning methods. Furthermore, we compared the spatial distributions of features extracted by a deep-learning model for each of four CV methods to help the intuitive understanding of the data leakage.

## Methods

### Participants

Seventy-seven PTSD patients and fifty-eight HCs were recruited from the Psychiatric Department of Inje University Paik Hospital. To evaluate psychiatric symptoms, three psychiatric symptoms were evaluated by clinical experts (Impact of Event Scale-Revises (IES-R) for stress level of traumatic events, Beck Depression Inventory (BDI) for depression level, and Beck Anxiety Inventory for anxiety level (BAI)). In addition, individuals without any psychiatric medical history were recruited for HCs from the local community. The demographic data and symptom scores of the participants are summarized in Table 1. The study was approved by the Institutional Review Board of Inje University Ilsan Paik Hospital (2015-09-018) and conducted in accordance with The Code of Ethics of the World Medical Association (Declaration of Helsinki), and all participants submitted written informed consent before the experiment.

**Table 1:** Demographic data of post-traumatic stress disorder (PTSD) patients and healthy controls (HCs).

	PTSD	HCs	*p*-value
Cases (*N*)	77	58	
Gender (male/female)	28/49	30/28	0.082
Age (years)	40.92 ± 11.93	39.98 ± 11.63	0.646
Education	13.51 ± 2.80	14.45 ± 3.37	0.120
IES-R	51.34 ± 21.71		
BDI	26.99 ± 13.13		
BAI	29.48 ± 15.44		

*PTSD* post-traumatic stress disorder, *HCs* healthy controls, *IES-R* impact of event scale-revised, *BDI* beck depression inventory, *BAI* beck anxiety inventory.

### EEG recording and preprocessing

Resting-state EEG data were recorded with a sampling rate of 1000 Hz for 5 min in eyes-closed condition, for which 64 Ag/AgCl electrodes were evenly mounted on the scalp according to the extended international 10–20 system (NeuroScan SynAmps2 (Compumedics USA, El Paso, TX, USA); references: both mastoids). Eye-related artifacts, such as blinks and movements, were removed by a regression approach based on mathematical procedures implemented in Curry 7, and gross-artifacts, i.e., motion (head and body movements) and muscle activities, were removed via visual inspection by experts. After that, artifact-free EEG data of 60 s extracted from task onset time point (eyes-closed condition) were used for further analysis because the shortest length of remaining EEG data was 60 s after the artifact removal among all participants. The EEG data were downsampled to 200 Hz to improve computational efficacy while keeping the most EEG frequency components below 100 Hz^13^; the training time for both deep-learning models was significantly reduced by more than 3 times when utilizing the downsampled EEG data compared to using the original EEG data (not shown here in detail). Then, to investigate the impact of data augmentation with respect to different amount of data, 60-s EEG data of each participant were cropped using five different window lengths (5, 10, 15, 20, and 60 s) with non-overlap and 75% overlap, respectively. Note that use of a 60-s window means that we used the original data without data augmentation because we extracted a 60-s data for each participant. The amount of data (number of trials) is more augmented using a smaller window length than using a relatively larger window length. After cropping EEG data, each EEG segment is regarded as a single trial.

### Cross-validation strategies

To investigate the impact of correct and incorrect CVs on the diagnostic performance of a CAD system after the data augmentation, we employed four different types of CV strategies that are sCV, osCV, tCV, and otCV, respectively. In case of sCV, all augmented trials of a single participant were used as either training data or test data, meaning that the whole trials of a participant were used together as a group when dividing the augmented trials of all participants into training and test data (correct CV). To calculate the diagnostic performance of a CAD system when using sCV, a voting strategy was employed with a threshold of 60% where we regarded a single participant as a correctly classified participant when more than 60% augmented trials of the participant were correctly classified^14^. The reason to introduce the voting method was because the goal of a CAD system is to diagnose a participant and thus a single diagnosis output should be provided by a CAD system for each participant. To investigate the impact of the number of trials on classification accuracy, the sCV strategy was also applied to trials augmented with a 75% overlap, referred to as overlapped sCV (osCV). On the other hand, in case of tCV, the augmented trials of a single participant were randomly divided into training and test data, and the diagnostic performance was estimated without a voting strategy as did in previous studies^15,16^. To further investigate the impact of the data leakage problem in tCV on diagnostic performance, we additionally applied the tCV strategy to the trials augmented with 75% overlap, which was defined as overlapped tCV (otCV)^17,18^. Table 2 provides the numbers of trials before and after the data augmentation for each CV and window length, respectively. The original number of samples was 135, but it significantly increased after data augmentation. Leave-one-out CV (LOOCV) is suitable for a small number of trials, e.g., the original data, but LOOCV is not suitable for a large enough number of trials after data augmentation because it might cause an overfitting problem due to a large size of training data^19^. To prevent this issue and keep consistency in terms of data analysis, a 10 × 10-fold CV was performed to estimate the diagnostic performances of the four strategies (sCV, osCV, tCV, and otCV).

**Table 2:** Numbers of trials before and after data augmentation for each cross-validation and window length, respectively.

		Before augmentation	After augmentation			
		60 s	20 s	15 s	10 s	5 s
CV method	sCV/tCV	135	405	540	810	1620
	osCV/otCV	405	1215	1620	2430	4860

CV indicates cross-validation. *sCV* subject-wise CV, *osCV* overlapped subject-wise CV, *tCV* trial-wise CV, *otCV* overlapped trial-wise CV.

**Figure 1:**
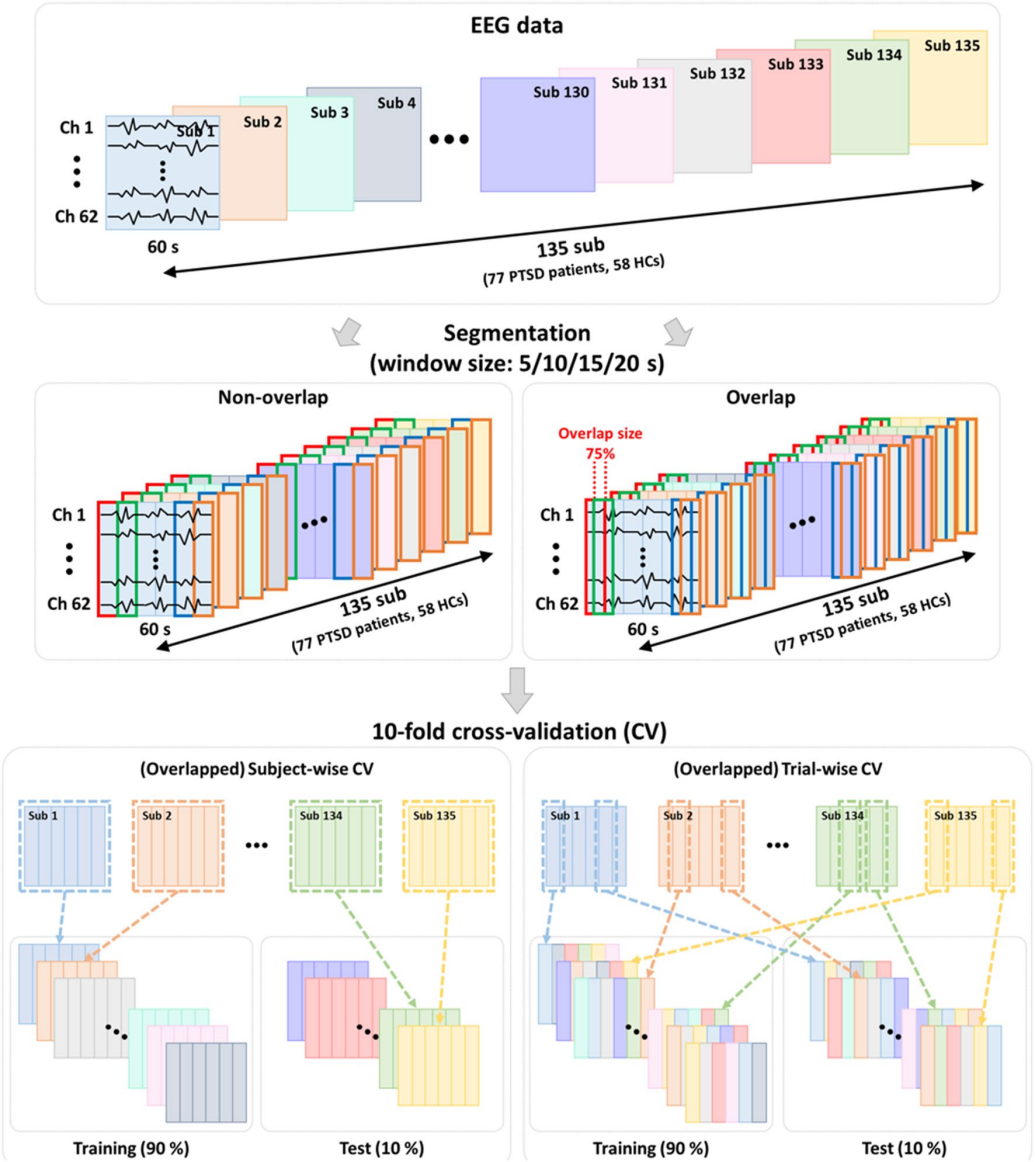
Two data augmentation approaches based on a cropping method (overlap and non-overlap) and four cross-validation (CV) strategies (subject-wise CV, overlapped subject-wise CV, trial-wise CV, and overlapped trial-wise CV). EEG data of all subjects (N = 135) are augmented by cropping the whole EEG data independently. Five different window lengths were used for data cropping to see the impact of the number of trials (5, 10, 15, 20, and 60 s) with non-overlap and overlap strategies, respectively. For subject-wise CV, all augmented trials of a single participant are used as either training or test data together, whereas augmented trials are randomly divided into training and test regardless of the participant for trial-wise CV. Overlapped subject-wise CV and overlapped trial-wise CV use a same approach to subject-wise CV and trial-wise CV, respectively, except that trials augmented based on an overlapped window are used. Note that different colors represent different participants.

Figure 1 represents the scheme of data augmentation approaches based on the cropping method and the four CV strategies (sCV, tCV, osCV, and otCV) used in the present study.

### Convolutional neural network (CNN) architectures

Recently, CNN-based deep-learning algorithms have been widely used to develop EEG-based CAD systems for psychiatric patients, and they showed high diagnostic performances in psychiatric research fields^3,20–22^. In the present study, two CNN models were used to evaluate the diagnostic performance: 13 layer-based CNN model (CNN-13)^22^ and EEGNet^23^ model. The architectures and parameters of CNN-13 and EEGNet are presented in Supplementary Figs. S1 and S2, respectively. CNN-13 was selected due to its high diagnostic accuracy (over 90%) for the discrimination of patients with depression and HCs using resting-state EEG data recorded from only two EEG channels^22^. CNN-13 model consisted of three different types of layers (5 convolutional layers, 5 pooling layers, and 3 fully-connected layers) where a leaky rectified linear unit (leaky ReLU) was used as an activation function and learning rate and dropout were set to 0.0001 and 0.1, respectively^22^. In addition, we tested another CNN-based model, namely EEGNet, which have shown excellent classification performances for time-series EEG data, to investigate the data leakage problem caused by an inappropriate CV regardless of deep-learning models^23^. EEGNet was composed of three different types of layers (3 convolutional layers, 2 pooling layers, and one fully-connected layer) where an exponential linear unit (ELU) was used as an activation function and learning rate and dropout rate were set to 0.001 and 0.5, respectively^23^. For both CNN models, we set a same batch size (= 5) and a same number of training epochs (= 300), respectively. In addition, loss was calculated based on a cross-entropy method and Adam optimizer was used to optimize the parameters of the CNN models, such as weights and learning rates. Finally, the diagnostic performance was computed using the balanced classification accuracy due to the imbalanced number of participants in each group (77 PTSD patients vs. 58 HCs).

### Feature distribution

Feature distribution was investigated to provide intuitive insights into the understanding of the data leakage problem, for which 992-dimensional features were extracted from the last convolutional layer of EEGNet for each of the four CV strategies when a window length of 10 s was used for data cropping as example feature distributions. To visually inspect the high-dimensional features, we reduced them into a two-dimensional domain using the t-stochastic neighbor embedding (t-SNE) method based on Euclidean distance^24^. In addition, a decision boundary was computed using linear discriminant analysis (LDA) based on training data^25^.

### Statistical analysis

**Figure 2:**
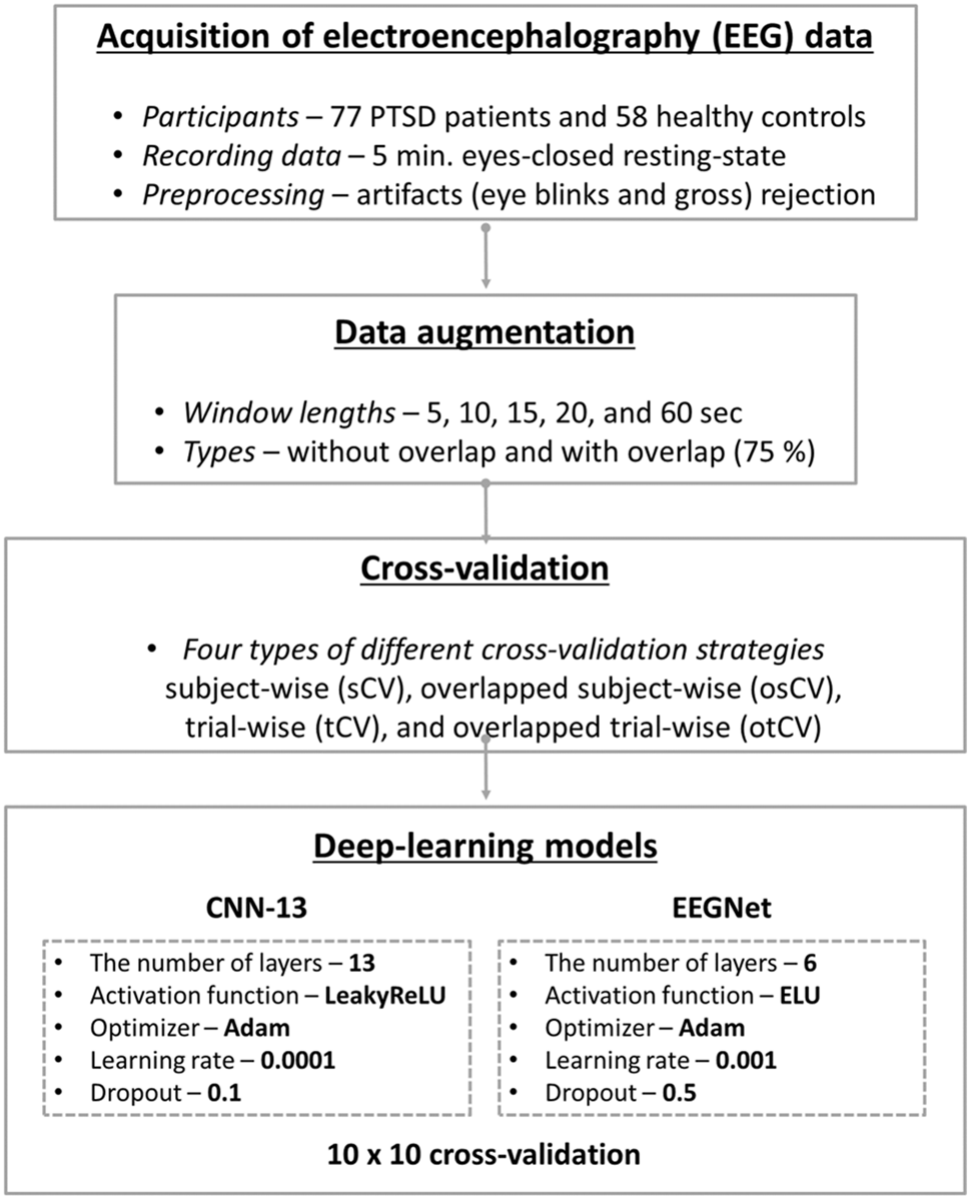
Flowchart of the deep-learning-based classification strategy.

To investigate the differences in diagnostic performances between the four different CV methods (sCV, osCV, tCV and otCV), statistical analysis was performed. To this end, Friedman’s test was conducted to evaluate the differences among the four CV methods for each window length (5, 10, 15, 20, and 60 s), and Wilcoxon rank sum test was used to evaluate the difference between two methods based on adjusted *p*-values using Bonferroni correction as a post-hoc analysis. All statistical tests were performed using MATLAB R2020b (MathWorks, Natick, MA, USA). Figure 2 presents the flowchart of the overall deep-learning-based classification analysis used in the present study.

## Results

### Classification accuracy

**Figure 3:**
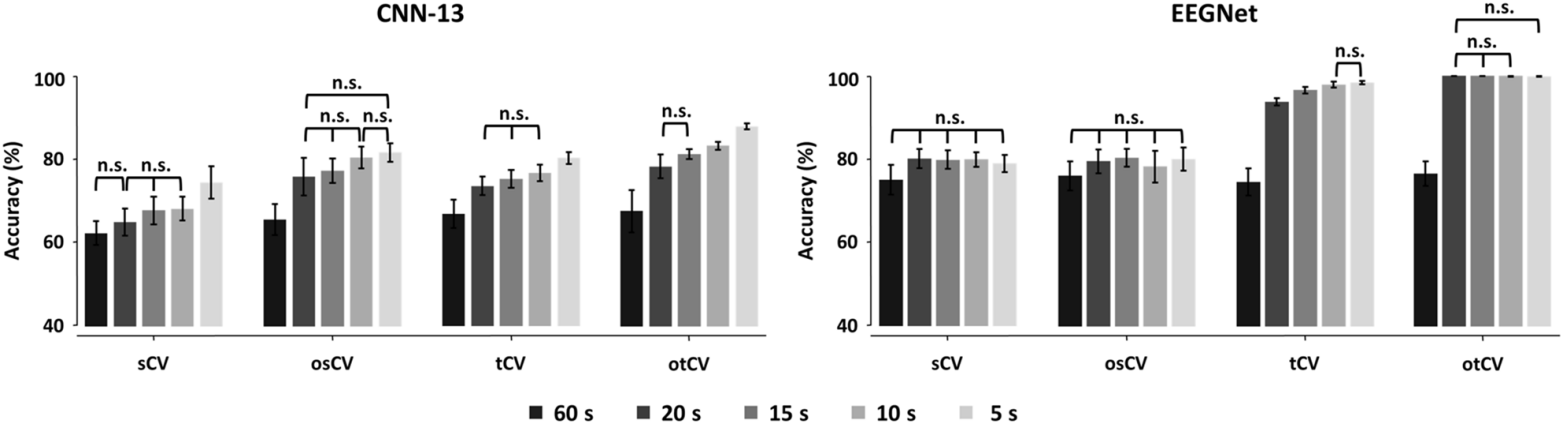
Mean and standard deviations of classification accuracies of different window lengths for two deep-learning models with respect to the cross-validation (CV) methods. Classification accuracies obtained using augmented data are significantly higher than that of the original data (60-s window length) in each CV method for the two deep-learning models (Bonferroni corrected *p* < 0.05), except for sCV method (20-s window length for CNN-13 and all window lengths for EEGNet) and for osCV method (all window lengths for EEGNet). n.s. means no significance, and the pairs without n.s. showed significant difference in terms of the classification performance.

Figure 3 shows the classification accuracies of the two deep-learning models for each window length used for data augmentation in terms of the CV method. For both deep-learning models, the classification accuracies tended to gradually increase as the window lengths decreased (increased data amount) regardless of the CV types, indicating that the cropping-based data augmentation can improve the overall diagnostic performances of the CAD system. In particular, the classification accuracies were significantly higher when using augmented data than when using the original data (60-s window) for all window lengths and CV method methods (Bonferroni corrected *p* < 0.05), except the 20-s window length for CNN-13 in sCV and all window lengths for EEGNet in both sCV and osCV. Table 3 provides the detailed classification accuracies for all window lengths and CV methods.

**Table 3:** Mean and standard deviations of classification accuracies for two deep-learning models with respect to the different window lengths for each CV method.

		60 s (original)	20 s	15 s	10 s	5 s
CNN-13	sCV	62.06 ± 2.86	64.77 ± 3.23	67.54 ± 3.34	67.96 ± 2.82	74.24 ± 3.94
	osCV	65.36 ± 3.80	75.70 ± 4.50	77.16 ± 2.93	80.33 ± 2.66	81.54 ± 2.25
	tCV	65.12 ± 3.50	71.98 ± 2.56	73.17 ± 2.59	74.19 ± 3.27	79.60 ± 1.83
	otCV	61.26 ± 4.54	80.36 ± 1.11	82.28 ± 1.02	83.61 ± 1.04	85.61 ± 0.65
EEGNet	sCV	74.79 ± 3.60	79.81 ± 2.33	79.57 ± 2.25	79.85 ± 1.41	78.72 ± 2.05
	osCV	75.83 ± 3.48	79.32 ± 2.89	80.13 ± 2.16	78.06 ± 3.79	79.86 ± 2.80
	tCV	74.30 ± 3.27	93.53 ± 0.85	96.38 ± 0.83	97.79 ± 0.46	98.12 ± 0.42
	otCV	76.41 ± 2.92	99.89 ± 0.09	99.92 ± 0.05	99.90 ± 0.03	99.77 ± 0.08

**Figure 4:**
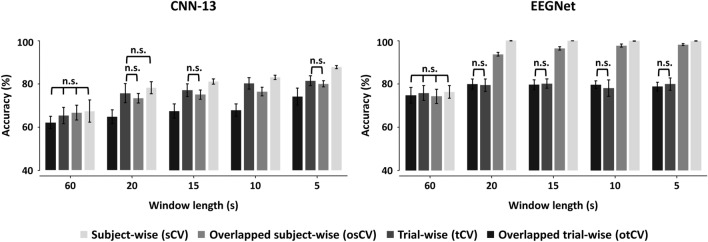
Mean and standard deviations of classification accuracies of four cross-validation (CV) strategies for two deep-learning models with respect to the different window lengths. For both deep-learning models, the classification accuracies of tCV and otCV are significantly higher than those of sCV for all window lengths, and those of otCV do than those of tCV, except the 60-s window length (original data) (Bonferroni corrected *p* < 0.05: otCV > tCV > sCV). In terms of osCV, the deeper model of CNN-13 shows improved classification performances compared to sCV, whereas the shallower model of EEGNet shows little changes in classification accuracy. n.s. means no significance, and the pairs without n.s. showed significant difference in terms of the classification performance.

Figure 4 shows the classification performances of the two deep-learning models for each CV method in terms of the window length where the same classification results shown in Fig. 3 and Table 3 were used. Note that when using original data without data augmentation (60-s window length), the four CV strategies were exactly same methods in terms of CV, but the slight difference of classification accuracies among the four CV strategies was derived from use of different training and test data randomly divided in each CV cycle; no significant difference between the CV strategies was observed for both deep-learning models when using the original data. For CNN-13 model, the classification accuracies of tCV and otCV were considerably higher than sCV by about 5–15%, respectively, across the different window lengths, and in particular the classification accuracies were more inflated for otCV than tCV. However, the classification performances of osCV were comparable to those of tCV and significantly higher than those of sCV (Bonferroni corrected *p* < 0.05), except for the absence of data augmentation (a 60-s window length), suggesting that osCV can improve classification performances without the issue of inflated performance for CNN-13. In particular, the classification accuracy of CNN-13 showed a gradual improvement as the number of trials increased, eventually surpassing 80% (81.54 ± 2.25%), which was significantly higher than the highest classification accuracy obtained using sCV for CNN-13 (74.24 ± 3.94%). These findings indicate that a relatively deeper model necessitates a larger training dataset to efficiently train the model. On the other hand, regarding EEGNet, both tCV and otCV showed significantly higher classification accuracies than those of sCV and osCV by about 15–20% when data augmentation was applied and otCV showed more inflated classification accuracies than tCV. However, unlike CNN-13, no notable increase in classification accuracy was observed for osCV compared to sCV, which would be caused by the fact that a relatively shallower model (EEGNet)^25,26^ can be effectively trained with a relatively smaller number of trials, thereby not showing further improvements in classification accuracy even with additional training data. Comparing the results of EEGNet with those of CNN-13, overall classification accuracy was higher for EEGNet when using a relatively smaller number of trials to train the model, but CNN-13 showed comparable or higher classification accuracy compared to EEGNet as the number of trials increased. Therefore, depending on the number of layers of deep-learning models, a suitable data augmentation strategy (either sCV or osCV) should be carefully considered to improve the classification performance as well as minimize computational time.

### Feature distribution

**Figure 5:**
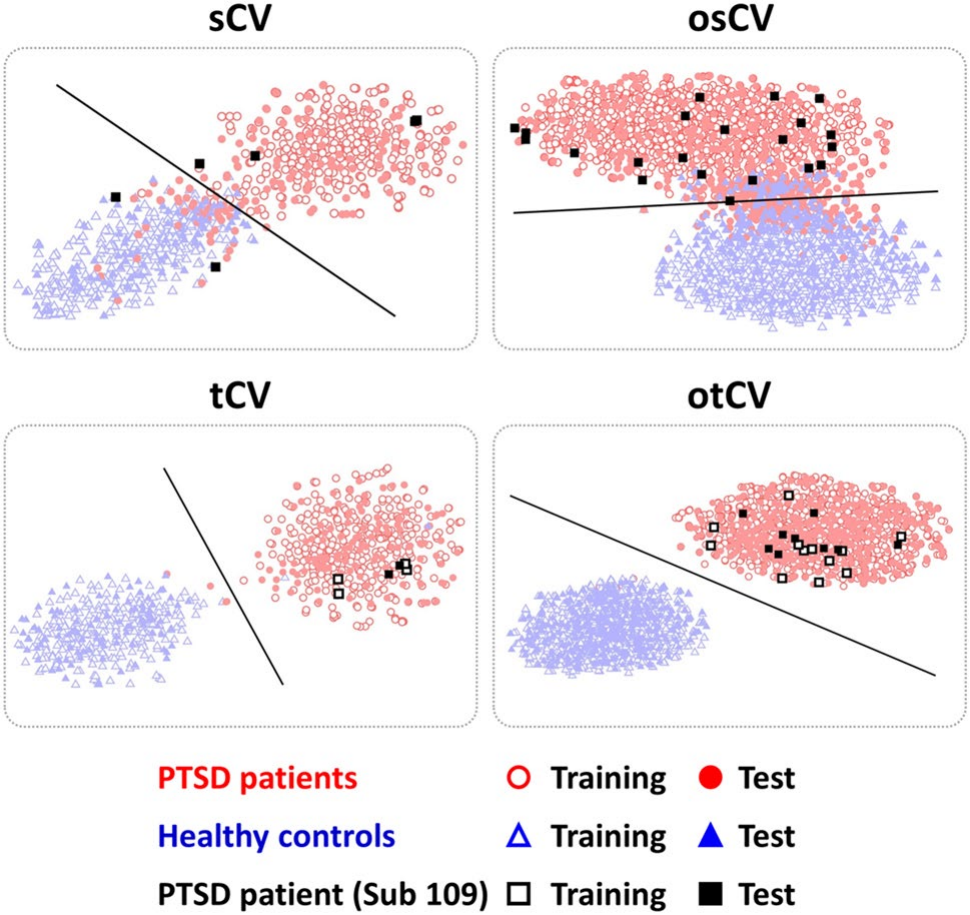
Distributions of the features extracted from EEGNet for each cross-validation (CV) strategy when a window length of 10 s was used. Because a 60-s EEG epoch was used for data analysis, use of the 10-s window length resulted in 6 trials after data augmentation for both subject-wise CV (sCV) and trial-wise CV (tCV), and 21 trials for overlapped sCV (osCV) and overlapped tCV (otCV), respectively. Red circles, blue triangles, black rectangles represent the features extracted from PTSD patients, healthy controls (HCs), and an example participant (#109 PTSD patient), respectively. Empty and filled symbols represent training and test data, respectively. The features of PTSD patients and HCs are independently clustered with some overlap between the two groups for sCV and osCV while those are more independently and densely clustered with little overlap between the two groups for tCV and otCV than for sCV and osCV because tCV and otCV used trials augmented from a same participant for both training and test data simultaneously. otCV shows better separability than tCV due to more data leakage.

Figure 5 represents the feature distributions of the four CV strategies with the LDA hyperplane in 2D t-SNE space. Red circles and blue triangles indicate the features extracted from PTSD patients and HCs, respectively. Empty and filled symbols show training and test data, respectively, and a solid line represents the LDA hyperplane separating PTSD patients and HCs. For sCV and osCV, the features of PTSD patients and HCs were independently clustered, but some overlap between the two groups were observed regardless of training and test data, whereas those were more independently and densely clustered with nearly perfect separation for both tCV and otCV, but otCV showed a better separation between two clusters (groups) than tCV. This result can be explained by the fact that trials augmented from a single participant were used for both training and test data simultaneously for tCV and otCV; the trials augmented from a single participant have similar feature values and they are naturally more clustered than those augmented from different subjects. This phenomenon is well represented with the augmented trials of an exemplary participant denoted by black rectangles in Fig. 5. For sCV and osCV, all rectangles are filled, meaning that all augmented trials for a specific participant were used as either training or test data. In this example, all trials were used as test data and this participant was classified into PTSD according to the thresholding-based voting method even though two of five trials were classified into HCs for sCV. On the other hand, for tCV and otCV, both empty and filled black rectangles were observed, indicating that the trials of a specific participant were used for both training and test data simultaneously. In particular, all test trials of a participant were classified into one class (PTSD) where all training trials extracted from the same participant were also observed in the same class; data leakage occurred between training and test data, leading to inflated classification performances.

## Conclusion

Use of data augmentation has been increasing with the significant advancement of deep-learning algorithms to increase their performance because many data are generally required to well train deep-learning models. In this study, we investigated the effect of different CV strategies on the performance of EEG-based CAD systems in the context of data augmentation. We showed that data augmentation could enhance the performance of deep-learning-based CAD systems (Table 2), but the classification performance could be significantly inflated when applying a wrong CV due to data leakage, hindering the accurate diagnosis of psychiatric disorders. Therefore, a correct CV method should be used to prevent the overly estimated classification performances due to data leakage by completely separating training and test data after data augmentation. As shown in this study, sCV with a voting strategy can be one of the solutions to obtain the accurate classification performance of CAD systems after data augmentation. This study provides a good guideline for researchers who are not familiar with data augmentation as well as deep-learning-based approaches when developing neuroimaging-based CAD systems for psychiatric disorders.

### Supplementary Information

Supplementary Information.

## Data Availability

The datasets used and/or analysed during the current study available from the corresponding author on reasonable request.
