# Publisher Correction: Risk of data leakage in estimating the diagnostic performance of a deep-learning-based computer-aided system for psychiatric disorders

**DOI:** 10.1038/s41598-024-52295-x

**Published:** 2024-01-25

**Authors:** Hyung-Tak Lee, Hye-Ran Cheon, Seung-Hwan Lee, Miseon Shim, Han-Jeong Hwang

**Affiliations:** 1 Department of Electronics and Information Engineering, Korea University, Sejong, Republic of Korea; 2 Interdisciplinary Graduate Program for Artificial Intelligence Smart Convergence Technology, Korea University, Sejong, Republic of Korea; 3 Psychiatry Department, Ilsan Paik Hospital, Inje University, Goyang, Republic of Korea; 4 Clinical Emotion and Cognition Research Laboratory, Goyang, Republic of Korea; 5 Department of Artificial Intelligence, Tech University of Korea, Siheung, Republic of Korea

Correction to: *Scientific Reports* 10.1038/s41598-023-43542-8, published online 03 October 2023

In the original version of this Article, the equal contribution status of authors Hyung-Tak Lee and Hye-Ran Cheon was inadvertently omitted.

The original Article has been corrected.

