# Supplementary material for: Risk of data leakage in estimating the diagnostic performance of a deep-learning-based computer-aided system for psychiatric disorders
Source: Sci Rep. 2023 Oct 3;13:16633. doi: 10.1038/s41598-023-43542-8 (PMC10547830; doi:10.1038/s41598-023-43542-8)
Supplement: Supplementary file 1 — Supplementary Information. [file 41598_2023_43542_MOESM1_ESM.docx]

**Supplementary Figure S1**. Architecture and parameters of 13 layers-based convolutional neural network (CNN-13).


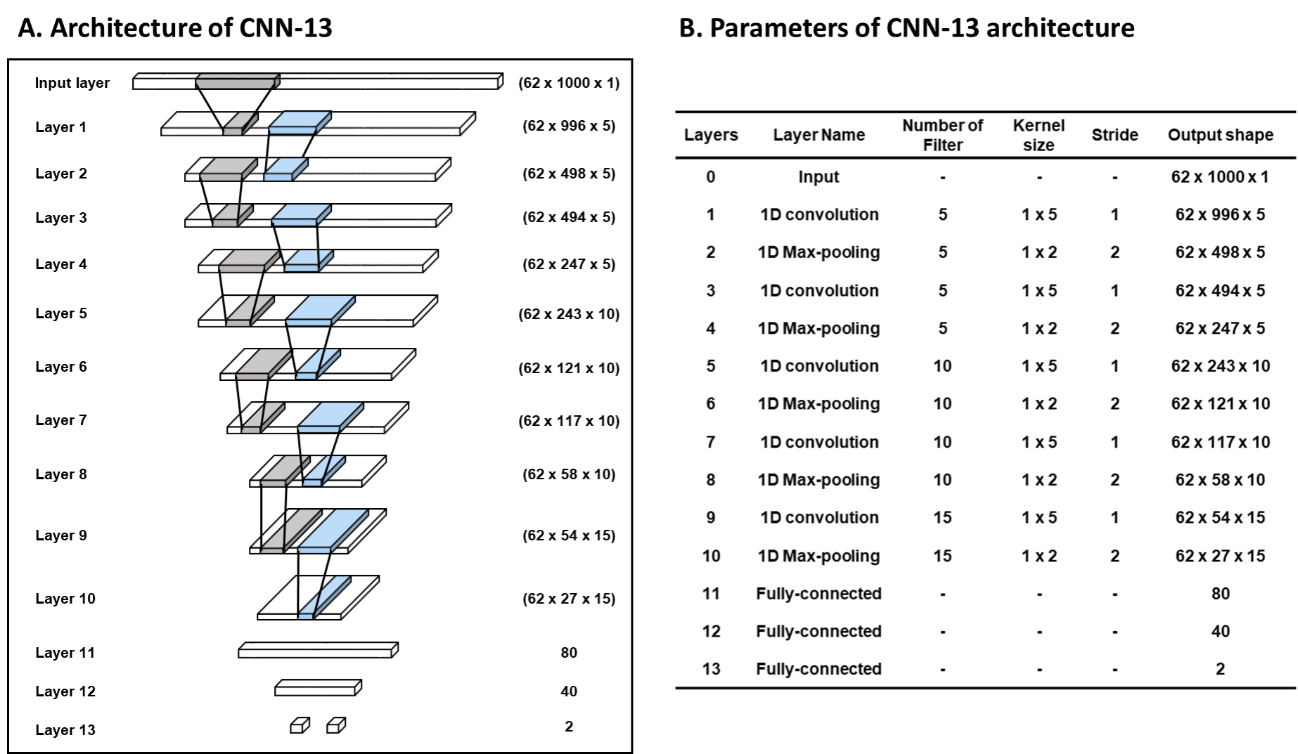


**Supplementary Figure S2.** Architecture and parameters of EEGNet.


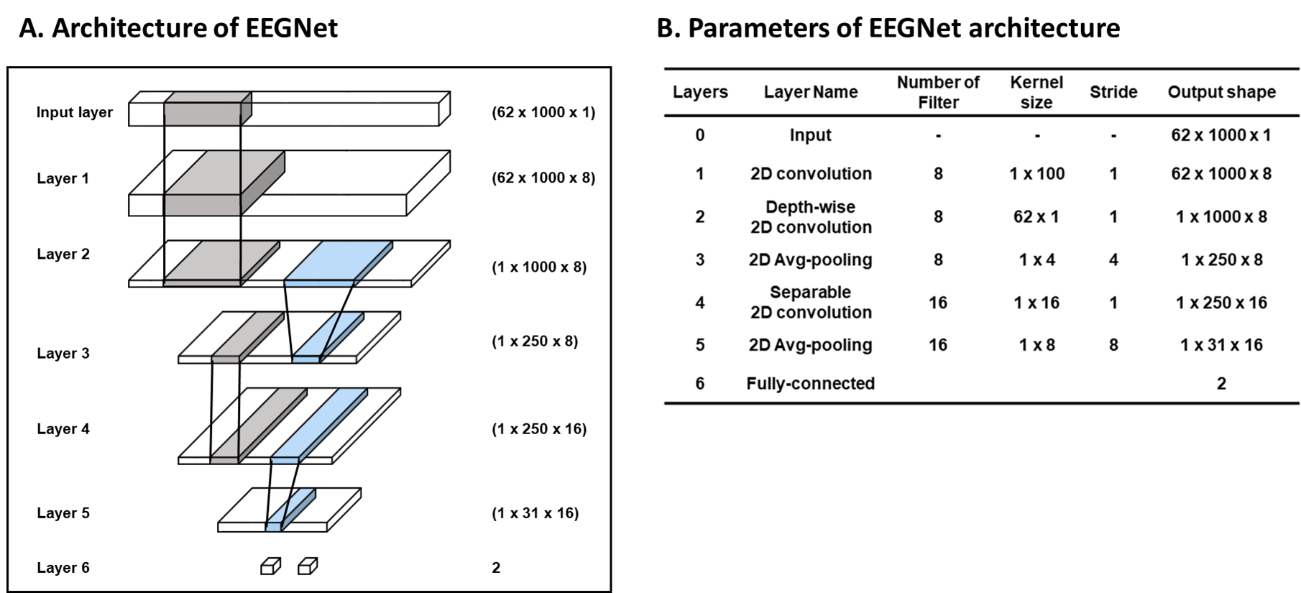


| **Supplementary Table S1.** True positive rate for the validation and test data, respectively. | | | | | | | |
| --- | --- | --- | --- | --- | --- | --- | --- |
|  | | | 60 s | 20 s | 15 s | 10 s | 5 s |
| CNN-13 | sCV | Validation | 78.82±2.77 | 83.98±3.95 | 83.07±3.87 | 81.23±3.97 | 78.45±4.46 |
|  |  | Test | 79.63±4.71 | 84.00±6.74 | 80.71±4.89 | 78.45±5.52 | 75.98±3.41 |
|  | osCV | Validation | 80.53±4.55 | 74.47±4.26 | 73.17±3.66 | 72.13±3.96 | 72.41±3.60 |
|  |  | Test | 79.93±4.67 | 70.96±3.72 | 70.59±3.34 | 71.00±4.03 | 69.95±2.07 |
|  | tCV | Validation | 79.35±3.07 | 77.28±3.04 | 75.12±1.73 | 75.89±2.01 | 75.22±1.42 |
|  |  | Test | 77.25±6.12 | 73.45±3.73 | 74.34±3.16 | 75.56±1.74 | 74.64±1.83 |
|  | otCV | Validation | 82.12±3.12 | 75.91±2.10 | 78.32±1.35 | 76.99±1.56 | 86.52±1.63 |
|  |  | Test | 81.59±4.31 | 78.72±2.67 | 76.42±1.60 | 76.44±1.46 | 85.96±1.94 |
| EEGNet | sCV | Validation | 83.88±4.01 | 77.65±4.05 | 79.22±4.49 | 76.06±6.87 | 75.69±5.60 |
|  |  | Test | 76.84±2.33 | 75.98±3.94 | 74.07±4.14 | 74.27±3.24 | 71.38±2.72 |
|  | osCV | Validation | 82.12±3.52 | 80.02±4.38 | 79.98±5.62 | 79.82±6.02 | 77.97±5.01 |
|  |  | Test | 75.54±5.13 | 74.61±3.54 | 78.23±4.80 | 73.43±4.33 | 74.25±4.19 |
|  | tCV | Validation | 82.53±2.48 | 94.73±0.78 | 96.42±1.32 | 97.68±1.09 | 97.98±0.34 |
|  |  | Test | 75.96±4.96 | 92.17±1.09 | 95.39±1.44 | 97.25±0.94 | 97.64±0.68 |
|  | otCV | Validation | 82.18±2.77 | 99.84±0.11 | 100.00±0.00 | 99.92±0.06 | 99.67±0.07 |
|  |  | Test | 77.09±2.62 | 99.86±0.15 | 99.85±0.07 | 99.85±0.05 | 99.64±0.12 |

| **Supplementary Table S2.** True negative rate for the validation and test data, respectively. | | | | | | | |
| --- | --- | --- | --- | --- | --- | --- | --- |
|  | | | 60 s | 20 s | 15 s | 10 s | 5 s |
| CNN-13 | sCV | Validation | 50.13±5.32 | 39.58±9.43 | 45.47±8.98 | 47.27±6.76 | 57.08±8.10 |
|  |  | Test | 44.50±6.56 | 45.53±10.05 | 54.37±10.18 | 57.47±8.22 | 72.50±7.71 |
|  | osCV | Validation | 48.13±8.02 | 68.93±7.67 | 74.97±7.42 | 78.95±6.71 | 79.84±4.61 |
|  |  | Test | 50.80±7.64 | 80.43±10.44 | 83.73±8.22 | 89.67±7.29 | 93.13±4.04 |
|  | tCV | Validation | 54.25±5.66 | 72.76±6.66 | 77.52±4.34 | 80.38±4.58 | 85.68±2.30 |
|  |  | Test | 56.10±7.57 | 73.32±6.55 | 75.93±4.78 | 77.55±4.50 | 85.57±2.94 |
|  | otCV | Validation | 47.63±5.67 | 78.81±6.45 | 86.97±2.69 | 90.00±2.34 | 90.34±1.94 |
|  |  | Test | 53.13±7.85 | 77.64±6.96 | 85.96±3.24 | 89.90±2.83 | 89.81±1.93 |
| EEGNet | sCV | Validation | 80.50±4.79 | 86.33±3.68 | 82.19±2.86 | 90.63±3.88 | 88.51±3.43 |
|  |  | Test | 72.73±6.03 | 83.63±3.49 | 85.07±2.63 | 85.00±3.57 | 86.07±3.54 |
|  | osCV | Validation | 78.00±5.72 | 84.83±3.99 | 83.88±3.62 | 88.63±2.32 | 83.05±2.96 |
|  |  | Test | 76.13±4.34 | 84.03±3.99 | 82.03±7.07 | 82.70±4.73 | 85.47±3.43 |
|  | tCV | Validation | 80.63±1.79 | 97.55±1.29 | 98.02±1.19 | 99.16±0.29 | 98.79±0.29 |
|  |  | Test | 72.63±7.37 | 94.90±1.30 | 97.38±1.30 | 98.10±0.90 | 98.61±0.43 |
|  | otCV | Validation | 78.63±4.43 | 100.00±0.00 | 99.99±0.02 | 99.96±0.06 | 99.91±0.07 |
|  |  | Test | 75.73±5.78 | 99.92±0.13 | 99.99±0.04 | 99.96±0.04 | 99.89±0.09 |

| **Supplementary Table S3.** F1 score for the validation and test data, respectively. | | | | | | | |
| --- | --- | --- | --- | --- | --- | --- | --- |
|  | | | 60 s | 20 s | 15 s | 10 s | 5 s |
| CNN-13 | sCV | Validation | 77.67±1.79 | 78.89±1.37 | 79.41±1.53 | 78.56±1.96 | 78.83±1.61 |
|  |  | Test | 71.42±2.95 | 74.59±2.75 | 74.75±2.15 | 74.01±2.57 | 76.47±3.80 |
|  | osCV | Validation | 78.47±2.45 | 78.58±2.08 | 78.81±1.59 | 78.96±1.67 | 79.37±2.05 |
|  |  | Test | 73.36±3.00 | 75.45±3.30 | 76.20±2.43 | 78.10±2.27 | 78.85±2.20 |
|  | tCV | Validation | 78.84±1.98 | 77.20±0.97 | 77.06±1.45 | 79.84±1.07 | 81.12±0.81 |
|  |  | Test | 72.78±3.72 | 75.79±1.44 | 77.09±2.00 | 78.51±1.22 | 80.37±1.26 |
|  | otCV | Validation | 79.40±1.88 | 79.46±0.76 | 83.45±0.71 | 83.12±0.55 | 89.23±0.70 |
|  |  | Test | 75.03±4.26 | 80.56±1.60 | 81.67±0.76 | 82.94±0.65 | 88.69±0.79 |
| EEGNet | sCV | Validation | 86.70±2.13 | 83.94±2.56 | 84.03±2.80 | 83.46±4.46 | 82.99±3.56 |
|  |  | Test | 77.24±2.82 | 79.84±2.59 | 79.01±3.09 | 79.28±1.97 | 77.60±1.65 |
|  | osCV | Validation | 84.99±2.62 | 85.06±3.00 | 84.71±3.62 | 85.61±3.90 | 83.31±3.57 |
|  |  | Test | 77.42±4.17 | 78.77±3.11 | 80.93±1.62 | 77.78±3.92 | 79.18±3.40 |
|  | tCV | Validation | 85.98±1.67 | 96.27±0.48 | 97.30±1.00 | 98.47±0.72 | 98.55±0.21 |
|  |  | Test | 76.45±3.10 | 93.99±0.75 | 96.62±0.79 | 97.87±0.72 | 98.28±0.39 |
|  | otCV | Validation | 85.29±1.61 | 99.92±0.06 | 100.00±0.01 | 99.95±0.04 | 99.80±0.04 |
|  |  | Test | 78.39±1.85 | 99.90±0.09 | 99.92±0.04 | 99.91±0.03 | 99.78±0.07 |

| **Supplementary Table S4.** Cohen’s kappa for the validation and test data, respectively. | | | | | | | |
| --- | --- | --- | --- | --- | --- | --- | --- |
|  | | | 60 s | 20 s | 15 s | 10 s | 5 s |
| CNN-13 | sCV | Validation | 28.50±5.05 | 24.14±6.36 | 27.98±6.14 | 27.97±4.68 | 33.90±3.44 |
|  |  | Test | 24.41±5.78 | 29.76±6.46 | 34.82±6.53 | 35.62±5.14 | 47.56±7.54 |
|  | osCV | Validation | 28.35±6.58 | 39.93±4.88 | 43.77±3.63 | 46.20±3.16 | 47.36±3.41 |
|  |  | Test | 31.24±7.37 | 49.86±8.36 | 52.46±5.55 | 58.80±4.96 | 60.86±4.25 |
|  | tCV | Validation | 32.68±5.69 | 49.90±4.03 | 52.38±3.80 | 55.05±3.25 | 58.98±1.93 |
|  |  | Test | 33.73±6.84 | 45.97±4.24 | 49.47±4.06 | 52.31±3.69 | 58.74±2.82 |
|  | otCV | Validation | 29.70±4.84 | 53.45±4.31 | 63.72±1.96 | 65.86±1.18 | 76.08±1.48 |
|  |  | Test | 35.09±10.11 | 55.66±5.45 | 60.98±2.29 | 64.65±1.71 | 75.03±1.46 |
| EEGNet | sCV | Validation | 62.02±4.96 | 59.22±4.97 | 57.86±5.03 | 61.10±6.77 | 58.75±5.63 |
|  |  | Test | 49.33±6.97 | 58.58±4.87 | 57.93±4.77 | 58.05±3.69 | 55.85±4.09 |
|  | osCV | Validation | 57.98±6.96 | 60.98±6.16 | 60.33±6.30 | 64.06±7.30 | 57.19±6.26 |
|  |  | Test | 51.06±7.14 | 57.41±5.77 | 59.43±3.84 | 55.01±7.74 | 58.50±5.47 |
|  | tCV | Validation | 60.43±3.62 | 92.06±1.12 | 94.33±2.08 | 96.56±1.21 | 96.54±0.48 |
|  |  | Test | 48.16±6.29 | 86.54±1.71 | 92.38±1.72 | 95.17±1.45 | 96.05±0.89 |
|  | otCV | Validation | 58.31±4.44 | 99.81±0.13 | 99.99±0.02 | 99.88±0.08 | 99.54±0.09 |
|  |  | Test | 52.43±5.54 | 99.77±0.20 | 99.81±0.10 | 99.78±0.07 | 99.49±0.16 |
